# MreB Forms Subdiffraction Nanofilaments during Active Growth in Bacillus subtilis

**DOI:** 10.1128/mBio.01879-18

**Published:** 2019-01-29

**Authors:** Cyrille Billaudeau, Zhizhong Yao, Charlène Cornilleau, Rut Carballido-López, Arnaud Chastanet

**Affiliations:** a MICALIS, INRA, AgroParisTech, Université Paris-Saclay, Jouy-en-Josas, France; b Inovarion, Paris, France; Stanford University; Harvard University

**Keywords:** SIM, TIRF, cell shape, cell wall, cytoskeleton, filament, microscopy, MreB, polymer, helix, protein localization, superresolution

## Abstract

The actin-like MreB protein is a key player of the machinery controlling the elongation and maintenance of the cell shape of most rod-shaped bacteria. This protein is known to be highly dynamic, moving along the short axis of cells, presumably reflecting the movement of cell wall synthetic machineries during the enzymatic assembly of the peptidoglycan mesh. The ability of MreB proteins to form polymers is not debated, but their structure, length, and conditions of establishment have remained unclear and the subject of conflicting reports. Here we analyze various strains of Bacillus subtilis, the model for Gram-positive bacteria, and we show that MreB forms subdiffraction-limited, less than 200 nm-long nanofilaments on average during active growth, while micron-long filaments are a consequence of artificial overaccumulation of the protein. Our results also show the absence of impact of the size of the filaments on their speed, orientation, and other dynamic properties conferring a large tolerance to B. subtilis toward the levels and consequently the lengths of MreB polymers. Our data indicate that the density of mobile filaments remains constant in various strains regardless of their MreB levels, suggesting that another factor determines this constant.

## Abstract (precis)

The construction of the bacterial cell envelope is a fundamental topic, as it confers its integrity to bacteria and is consequently the target of numerous antibiotics. MreB is an essential protein suspected to regulate the cell wall synthetic machineries. Despite two decades of study, its localization remains the subject of controversies, its description ranging from helical filaments spanning the entire cell to small discrete entities. The true structure of these filaments is important because it impacts the model describing how the machineries building the cell wall are associated, how they are coordinated at the scale of the entire cell, and how MreB mediates this regulation. Our results shed light on this debate, revealing the size of native filaments in B. subtilis during growth. They argue against models where MreB filament size directly affects the speed of synthesis of the cell wall and where MreB would coordinate distant machineries along the side wall.

## INTRODUCTION

Determining the mechanisms of cell shape establishment is one of the key goals of cell biology. In bacteria, it is widely assumed that the extracytoplasmic cell wall (CW) is the major physical determinant of cell shape. This envelope is universally composed of peptidoglycan (PG), also named murein, a continuous three-dimensional polymeric network on which are anchored additional (but essential) components such as teichoic acids (TA) in Gram-positive bacteria or lipoproteins anchoring the outermost membrane in Gram-negative bacteria (1).

The composition and biosynthetic pathway of PG are well established, but its three-dimensional organization and the mechanism controlling its assembly remain mysterious (2). In rod-shaped bacteria, PG-synthesizing enzymes belong to two spatially distinct machineries: the divisome, in charge of synthesizing the division septum at mid cell, and the so-called PG elongation machineries (PGEMs) in charge of producing the lateral CW material. The PGEMs include some poorly characterized “Mre” (for murein region) and “Rod” proteins, including MreB, a bacterial homolog of actin, essential for the correct establishment of the rod shape (3). A distinctive feature of MreB proteins is their mobile nature, early documented in cellular biology studies using fluorescent fusions to the green fluorescent protein (GFP), in both Gram-positive and Gram-negative models (4–8). These observations were later refined using advanced techniques, total internal reflection fluorescence (TIRF) and confocal microscopy (9–11), demonstrating the circumferential motion of the protein. Recently, we found that MreB displays an even more complex dynamic behavior, with subpopulations of proteins exhibiting different dynamics in the cytoplasmic membrane: circumferentially moving, randomly diffusing, or exhibiting constrained diffusion (static or with limited movement) (12).

However, despite much research, MreB still lacks a proven function (3). The prevailing model is that MreB controls the motion of the PGEM (3, 13–15). In fact, it was shown that directionally moving MreB patches depend on PG synthesis and reflect that of the machineries (9, 10). Recent results suggest that the direction of MreB motion results from the intrinsic property of MreB polymers to align along the highest concave curvature in the cell (16). In bacteria with multiple MreB isoforms such as Bacillus subtilis (which also bears Mbl and MreBH), it is supposed that the control of cell shape is shared between the homologs (17), although the characteristic phenotype associated with each mutant suggests potentially some additional specific function(s) (3).

A unique characteristic of MreBs is their distinctive pattern of localization, originally described as helical-like (4–8). The complex and extended structures first observed *in vivo* were in agreement with concomitant *in vitro* studies showing micron-long polymers of purified MreB from the thermophilic bacterium Thermotoga maritima (18, 19), and long filaments observed later when various MreB proteins were expressed in different heterologous hosts (20–22). Controlled polymerization allowing the formation of extended filaments of MreB was, by analogy with eukaryotic actin, suggesting potential mechanisms for the movement of the PGEM, and could conveniently explain how PGEM would be synchronized to ensure a continuous error-proof synthesis along the side wall (23).

Because it was uncertain whether MreB filamentous structures are present in their native host (with the notable exception of the impressive figure 8's obtained with a YFP-MreB fusion in Escherichia coli in reference 24) and their dynamics are difficult to characterize with conventional imaging, improved microscopy approaches were employed. TIRF and confocal microscopy showed that in growing cells, MreB forms discrete light diffraction-limited assemblies (9–11), incompatible with the model of long-range MreB filaments spanning the length of the cell and potentially connecting and synchronizing PGEM. Importantly, MreB patches appear as spherical or elliptical foci close to the diffraction limit (∼300 nm) when observed using optical microscopes. This results from the physical limitations of conventional light microscopy and does not exclude a filamentous subdiffraction structure (12). The unambiguous long bright filaments of E. coli YFP-MreB have since been shown to be an artifact generated by the tag (25). However, the erroneous interpretation of other GFP-MreB localizations was probably multifactorial (for a discussion, see reference 3). Importantly, the usage of improved imaging techniques allowed the visualization of MreB during active CW extension (i.e., from early to mid-exponential growth), while in early studies cells were observed during late exponential growth when fluorescent intensity was more intense, and, as shown in reference 9, longer structures could be observed. Altogether, TIRF and confocal imaging revealed the formation of discrete patches along the plasmid membrane during active growth, while longer (but non-helical) structures appear in later growth phases (9).

More recently, however, two joint publications, using cutting edge superresolution microscopy (SIM-TIRF and STED) techniques claimed the existence of micron-long MreB filaments during active growth in B. subtilis (13, 26). Strikingly, one study also observed these long filaments in our B. subtilis strain expressing an inducible GFP-MreB fusion (RCL238), that in our hands formed diffraction-limited patches (12, 26). These reports opened the path for a revived model of long MreB filaments distributed in a helical pattern in order to synchronize PGEM during growth (23). This latest review opportunely highlighted that the size of MreB assemblies, their substructures *in vivo* during active growth, conditions potentially affecting their length and organization, and how all these factors contribute to their dynamics (speed and direction) remain uncertain.

Here, we aimed to clarify these questions by combining TIRF microscopy and superresolution structured illumination microscopy (SIM) to image native and inducible GFP-MreB fusions in B. subtilis cells in different phases of growth. Our results show that long filamentous MreB structures are a common feature of B. subtilis strains that artificially accumulate high levels of GFP-MreB when cells are no longer actively growing and enter the stationary phase of growth. At natural levels, MreB forms light diffraction-limited assemblies in all phases of growth, which were partially resolved by SIM revealing their elongated nanometric structure. We found that MreB nanofilaments are oriented perpendicularly to the long axis of the cell, regardless of their length or dynamic behavior. We also show that filament length and dynamic behavior are not correlated, suggesting that filament length purely reflects the cellular levels of MreB and has no regulation properties toward MreB function. Finally, our results suggest that MreB is in excess toward the PGEM and that this excess is buffered by formation of longer structures and nonmobile filaments.

## RESULTS

### MreB forms discrete patches during exponential growth in TIRFM.

The discrepancy between previous reports that MreB forms small, discrete patches close to the diffraction limit (9, 10, 12) and two publications reporting micron-long GFP-MreB filaments in B. subtilis cells suggested that some growth conditions or genetic backgrounds might prompt the formation of MreB assemblies of different lengths and that these could inform on the properties and potential functions of MreB proteins.

**FIG 1:**
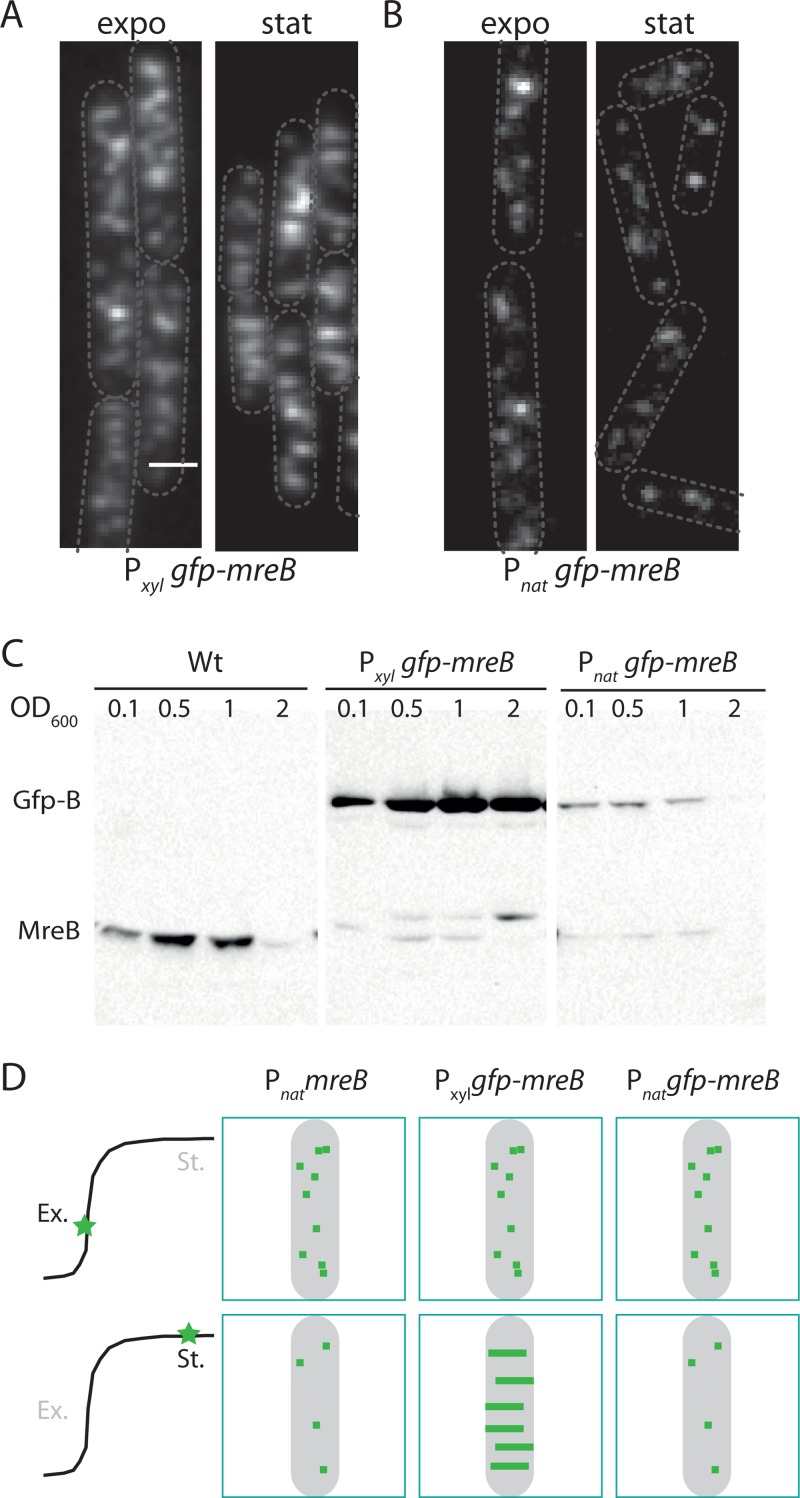
MreB forms extended structures during stationary phase. (A and B) TIRF acquisitions of B. subtilis strains expressing *GFP-mreB* under an inducible (RCL238) (A) or natural (NC103) (B) promoter, grown to exponential (expo) or stationary growth phase (stat) in the presence of 0.5% xylose. Bar = 1 µm. (C) Kinetics of accumulation of MreB in B. subtilis strains revealed by immunoblotting using anti-MreB antibodies. The strains are the wild-type strain 168 (wt), RCL238 (P*_xyl_ gfp-mreB*), and NC103 (P*_nat_ gfp-mreB*). Sampling of the cultures was performed at the indicated ODs (exponential growth typically ends at an OD of ∼0.6). (D) Schema summarizing the impact of the growth phase (exponential [Ex.] and stationary [St.] growth phase) (left drawing) and the genetic context on the appearance of short or long structures of MreB (green dots and lines).

We had never observed long MreB filaments in a variety of growth media in exponentially growing B. subtilis cells (9, 12), but we had noticed when cells entered stationary phase that an inducible GFP-Mbl fusion localized as transverse bands (9). Thus, we checked whether GFP-MreB was also able to form long filaments when cells exit exponential growth. We used our previously reported xylose-inducible GFP-MreB fusion in which GFP-MreB is the only copy of MreB present in the cell (RCL238) and a merodiploid strain expressing a GFP-MreB fusion under a xylose-inducible promoter in addition to its native *mreB* gene at its natural locus (JS17, a kind gift from P. Graumann, Philipps Universität, Marburg, Germany) (see Table S1 in the supplemental material). We imaged cells in LB medium from a very low density up to 2 h after cells had reached stationary phase of growth, in the presence of 0.5% xylose, a concentration of inducer allowing the levels of GFP-MreB produced in this strain to be similar to that of the wild-type MreB levels during exponential growth (9). In exponentially growing cells (OD of ∼0.2), inducible GFP-MreB fusions formed small, light diffraction-limited entities, as we reported previously (Fig. 1A; see also Fig. S1A and Movie S1 in the supplemental material). However, longer assemblies progressively appeared from the transition from exponential to stationary phase of growth, culminating with long filaments in deeper stationary phase, when cells are no longer actively growing (Fig. 1A, Fig. S1A and S1D, and Movie S1). Similar observations were made with the MreB paralog Mbl: an inducible GFP-Mbl fusion (as the only copy of Mbl in the cell; strain 2523) formed diffraction-limited patches during active exponential growth, while virtually all the GFP-Mbl assemblies formed long transverse bands spanning the cell diameter in stationary phase (Fig. S1B).

10.1128/mBio.01879-18.1 FIG S1 (A and B) TIRF acquisitions of B. subtilis strains expressing GFP-MreB (JS17) (A) or GFP-Mbl (2523) (B) under the control of a xylose-inducible promoter, grown to exponential (expo) or stationary (stat) growth phase in the presence of 0.5% xylose. Bar = 0.5 µm. (C) Kinetics of accumulation of MreB in B. subtilis strains revealed by immunoblotting using anti-MreB and anti-GFP antibodies. Strains are wild-type 168 (wt) or harbor xylose-inducible GFP-MreB fusions (JS17 and RCL238). Sampling of the cultures was performed at the indicated ODs (exponential growth typically ends at an OD of ∼0.6). (D) Typical growth curve of a wild-type B. subtilis 168 strain grown in rich LB medium at 37°C. The cell density, estimated as the optical density at 600 nm (blue), increases exponentially up to an OD of ∼0.5 to 0.6 (dotted line). During this period, the generation time (red) remains constant (generation time is calculated over a sliding 30 min window along the growth curve). After this point, cells enter an ∼80 min of complex transition phase due to diauxie, before reaching a plateau above an OD of ∼1.5. Download FIG S1, TIF file, 1.1 MB. Copyright © 2019 Billaudeau et al. 2019 Billaudeau et al. This content is distributed under the terms of the Creative Commons Attribution 4.0 International license.

10.1128/mBio.01879-18.4 TABLE S1 Strains used in this study. Download Table S1, TIF file, 0.2 MB. Copyright © 2019 Billaudeau et al. 2019 Billaudeau et al. This content is distributed under the terms of the Creative Commons Attribution 4.0 International license.

10.1128/mBio.01879-18.6 MOVIE S1 TIRF acquisitions of B. subtilis strains expressing *GFP-mreB* under an inducible promoter (RCL238), grown to exponential (Expo) or stationary (Stat) growth phase in the presence of 0.5% xylose. Images of cells in exponential and stationary phase were taken every second for 1 min and every other second for 2 minutes, respectively (thus, the “stat” record appears accelerated 2× compared to “expo”). Download Movie S1, AVI file, 0.1 MB. Copyright © 2019 Billaudeau et al. 2019 Billaudeau et al. This content is distributed under the terms of the Creative Commons Attribution 4.0 International license.

We concluded that in B. subtilis, diffraction-limited assemblies are the dominant form of MreB proteins during active growth and that the transition to stationary phase prompts the formation of long filaments by inducible fusions.

### Extended filaments of MreB appear when it accumulates above its native levels.

We noticed that at later stages of growth the intensity of membrane-associated MreB assemblies increased in the strains with inducible fusion, suggesting an accumulation of GFP-MreB. We hypothesized that these strains accumulate the GFP fusions during growth relative to native levels, potentially favoring the extension of the filaments. We first analyzed the total MreB content in B. subtilis strains grown from early exponential to late stationary phase by western blotting. In the wild-type strain, MreB levels peaked by the end of the exponential phase of growth (OD of 0.5) and dramatically decreased in late stationary phase (OD of 2). In sharp contrast, GFP-MreB accumulated during growth in the strains bearing the two xylose-inducible constructs (Fig. 1C; Fig. S1C and S1D). It should be noted that the high MreB levels reached during late stationary phase in these strains were never matched in the wild-type strain at any time of growth.

We concluded that *mreB* is the subject of regulation (either at the transcriptional or post-transcriptional level) that is lost when the *gfp-mreB* construct is placed under the control of an inducible promoter. In order to bypass this issue, we took advantage of the recently engineered NC103 strain in which *gfp-mreB* (the same fusion as in strain RCL238) was placed at its original locus, devoid of potentially interfering resistance marker and under its natural promoters (27). GFP-MreB levels assayed by western blotting revealed a drop similar to wild-type MreB when cells entered stationary phase (Fig. 1C), indicating that the stationary phase plateauing of GFP-MreB levels in strains RCL238 and JS17 was reflecting a transcriptional misregulation. Strikingly, TIRF imaging of NC103 cells revealed an absence of long filaments in both the exponential and stationary phases of growth (Fig. 1B), suggesting that MreB levels are the primary determinant of the formation of extended MreB filaments and that micron-long MreB filaments were an artifact of the accumulation of the fusion proteins above native levels (Fig. 1D).

### MreB forms ∼170 nm-long nanofilaments during active growth regardless of their dynamic behavior.

**FIG 2:**
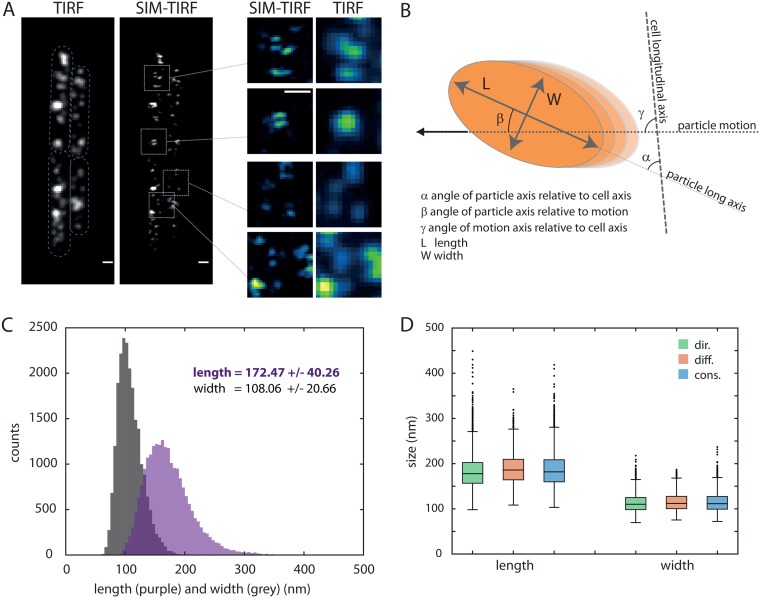
MreB forms nanofilaments oriented perpendicularly to the long axis of the cell. (A) Comparative observation of GFP-MreB localization (strain RCL238) in TIRF and SIM-TIRF modes. The TIRF image results from averaging the nine raw acquisition used for the SIM reconstruction. Bars, 0.5 µm. (B) The 2D Gaussian fit (G-Fit) method allows us to discriminate between a long (L) and short (w) axis for each particle. A threshold of L/W ≥ 1.4 is applied on subsequent analyses. The angle between the long axis of the filament and that of the cell (α) can be determined for all filaments regardless of their dynamic behavior. Angles β and γ (relative to the particle motion) can be calculated as well but for directionally moving filaments only. (C) Distribution of the length (purple) and width (gray) of GFP-MreB particles as determined by the G-fit method (see Materials and Methods). The length and width of each particle are the median values detected along their track. Indicated on the panel are the mean values ± standard deviations. Data were collected from populations of B. subtilis RCL238 cells grown to mid-exponential phase, expressing wild-type levels of GFP-MreB fusion. *n* = 22,053. (D) Distribution of the length and width of GFP-MreB particles for each subpopulation (dir., directed; diff., diffusive; cons., constrained), after classification using the MSD method (see Materials and Methods). Data were collected as described above for panel C. *n* = 4,858 (dir.), 1,154 (diff.), and 2,818 (const.).

Previous attempts to quantify B. subtilis polymer sizes *in vivo* gave mean lengths of 620 nm for MreB and 440 nm for Mbl filaments (13, 26), potentially suggesting an overaccumulation of these proteins at the time of observation. Because in our experiments, MreB forms discrete, close to or diffraction-limited patches during active growth, we therefore combined TIRF with superresolution structured illumination microscopy (SIM) in a SIM-TIRF setup allowing a lateral resolution of ∼110 nm (see Materials and Methods), to resolve its subdiffraction structure during active growth (28). We focused first on our strain expressing an inducible *gfp-mreB* (RCL238), in which MreB levels appear to be the closest to those in the wild-type at mid-exponential growth (Fig. 1C). While when examined in TIRF mode, MreB formed spherical patches of ∼250 to 300 nm in diameter, typical of particles close to or smaller than the diffraction limit as previously reported (12), when examined by SIM-TIRF, MreB appeared as significantly shorter and frequently anisotropic assemblies (Fig. 2A and Movie S2). We quantified the size and orientation of the patches using a customized 2D Gaussian fit (G-fit) method that determines the long and short axes of the assemblies regardless of their apparent movement (see Materials and Methods for details) (Fig. 2B). We found that 85.3% of all patches have a length/width ratio of >1.25, indicative of an anisotropic structure. Most lengths were above the resolution limit of the SIM-TIRF approach (98.1% > 110 nm; *n* = 22,053) and shorter than the field of view in the TIRF section (600 to 800 nm for a cell diameter of 1 µm and 100 to 200 nm TIRF penetration depth). We could therefore measure with confidence the length of MreB assemblies and found that they have a mean length of 172 nm ± 41 nm during exponential growth (Fig. 2C). The average width of the filaments (108 nm ± 21 nm) is necessarily overestimated since only 40.4% of the width values are above the resolution limit. Similar filament lengths were observed for other exponentially growing strains expressing inducible and native GFP-MreB or GFP-Mbl fusions (Fig. S2D).

10.1128/mBio.01879-18.2 FIG S2 (A) Length quantification of long filaments using a kymograph. In panel 1, by TIRF or SIM-TIRF, directionally moving MreB particles appear as discrete, light diffraction-limited dots (a) or filamentous structures (b). In panel 2, profile lines (1-pixel width) were drawn along separated filament trajectories (red lines) in order to generate kymographs. In panel 3, on kymographs, slices are piled up vertically: the distance (*d*) and time (*t*) are on horizontal and vertical axes, respectively. Punctuated structures appear as lines, while elongated structures appear as parallelograms. In panel 4, velocity (*v*) can be estimated as the angle of the lines (for discrete structures) or the parallelograms (for filamentous structures). *t*_vis_ corresponds to the time between the appearance of both extremities of a filament at a given position. For accuracy, we averaged the *t*_vis_ from each position along *d*. The length of filaments was estimated as *v*×*t_vis_*. In panel 5, only filaments entirely visible (green) during the window of acquisition (and excluding the first and last five frames of movies for certainty) were kept. (B) Kymograph and 2D Gaussian fit methods give similar length measurements for short structures. Distribution of length of directed GFP-MreB fusions in B. subtilis strain (RCL238) grown to exponential phase, quantified using the kymograph (green; *n*= 218) or the 2D Gaussian fit (gray; *n* = 31,376) method. (C) Determination of the length of simulated filaments shows the requirement of a kymograph approach to measure long structures. Objects ranging from 100 to 1,300 nm long were simulated and measured using three methods: kymograph (black), 1D Gaussian fit of intensity profile (red), or 1D Gaussian fit of intensity profile with a simulated intensity decrease due to TIRF illumination (magenta). For this, a correction factor to the intensity exp(−*z/d*) is calculated, allowing us to take into account penetration depth in TIRF (*d*=250 nm) and height *z* of filament relative to the coverslip, where *r* is the cell radius and *dx* is the lateral distance separating the filament position from the contact point of the cell with the coverslip (inset). Error bars correspond to standard deviations (*n* = 30). The blue dashed-dotted line corresponds to cell diameter. The black dashed-dotted line shows *y = x*. (D) Distribution of length of GFP-MreB (strains RCL238, JS17, and NC103) and Mbl-GFP (strain 2521) fusions in B. subtilis strains during exponential growth. The lengths shown are the median values detected along their track and were determined by the G-fit method. *n* = 13,070 (NC103), 22,053 (RCL238), 13,666 (JS17), and 4,625 (2521). (E) Distribution of length of GFP-MreB (RCL238, JS17, and NC103) and Mbl-GFP (2521) fusions in B. subtilis strains during stationary phase. Lengths are estimated on directionally moving subpopulation of filaments by the kymograph method (see Materials and Methods). *n* = 37 (RCL238), 35 (JS17), 38 (NC103), and 85 (2521). (F) Example of micron-long GFP-MreB artifacts forming during stationary phase. Cells of the merodiploid JS17 strain were grown in rich LB medium and observed 2 h after entry into stationary phase. A slice of a 3D SIM-TIRF acquisition corresponding to Movie S3 is shown here. Download FIG S2, TIF file, 0.6 MB. Copyright © 2019 Billaudeau et al. 2019 Billaudeau et al. This content is distributed under the terms of the Creative Commons Attribution 4.0 International license.

10.1128/mBio.01879-18.7 MOVIE S2 TIRF and SIM-TIRF comparison of acquisitions on B. subtilis strains expressing *GFP-mreB* under an inducible promoter (RCL238), grown to exponential phase in the presence of 0.5% xylose. Images were taken every second for 1 min and processed with false color to enhance the visualization. Bars = 0.5 µm. Download Movie S2, AVI file, 0.3 MB. Copyright © 2019 Billaudeau et al. 2019 Billaudeau et al. This content is distributed under the terms of the Creative Commons Attribution 4.0 International license.

We recently reported that MreB patches coexist as three classes with different dynamic behaviors in the cell: constrained, randomly, or directionally moving (12). Therefore, we wondered whether a correlation may exist between filament dynamics and filament dimensions. Using our previously published method for MreB single particle analysis (12), we quantitatively scrutinized the set of SIM-TIRF acquired data, sorted the particles based on their dynamic behavior, and determined the distribution of lengths and widths of each subpopulation (Fig. 2D). No significant difference of either MreB filament length or width was detected, suggesting that they have no influence on the dynamic properties.

We also determined the lengths of the extended filaments observed during stationary phase, although they do not reflect physiological structures. For this, we used exclusively the kymograph analysis method (see Materials and Methods), because the longest filaments observed under this condition extend beyond the visible field of view of the TIRF section and their size would be underestimated by the G-fit method (see Materials and Methods; Fig. S2C). As expected, the filaments were on average two times longer in the two inducible strains and presented a broader distribution of length during stationary phase than during exponential growth (Fig. S2E). These assemblies could reach a maximum close to a micron long, reminiscent of previously published filamentous structures spanning the cell diameter observed by conventional epifluorescence microscopy and more recently with SIM and STED approaches (4–8, 13, 26). The longest structures were observed in the inducible merodiploid strain JS17 (Fig. S2E and S2F and Movie S3), consistent with the more important accumulation of total MreB during growth in this strain (Fig. S1C).

10.1128/mBio.01879-18.8 MOVIE S3 Example of micron-long GFP-MreB artifacts forming ring-like structures during stationary phase. Cells of the merodiploid JS17 strain were grown in rich medium and observed 2 h after entry into stationary phase. A 3D reconstruction of a 3D SIM-TIRF acquisition is presented here. Download Movie S3, AVI file, 2.0 MB. Copyright © 2019 Billaudeau et al. 2019 Billaudeau et al. This content is distributed under the terms of the Creative Commons Attribution 4.0 International license.

### MreB nanofilaments are oriented perpendicularly to the long axis of the cell regardless of their dynamic behavior.

The long filaments of MreB previously studied were shown to be oriented perpendicularly to the long axis of the cell (13, 26), thus roughly in the direction of the directionally moving MreB filaments (12), and Olshausen and collaborators showed that extended MreB filaments propagated along their main axis (13). We decided to assess whether the orientation of MreB nanofilaments during active growth correlates with their trajectories. Furthermore, recent work from Hussain et al. showed that MreB filaments orient along the higher curvature of the cell, that is perpendicularly to the long axis of the cell, in order to minimize the energy due to the interaction between the bent cell membrane and the more highly bent MreB polymers (16). We thus wondered whether longer filaments were more constrained in the direction of the highest curvature because of the greater surface of interaction between the polymer and the membrane and, conversely, whether smaller particles might be less prone to align around the mean angle α of 90°.

**FIG 3:**
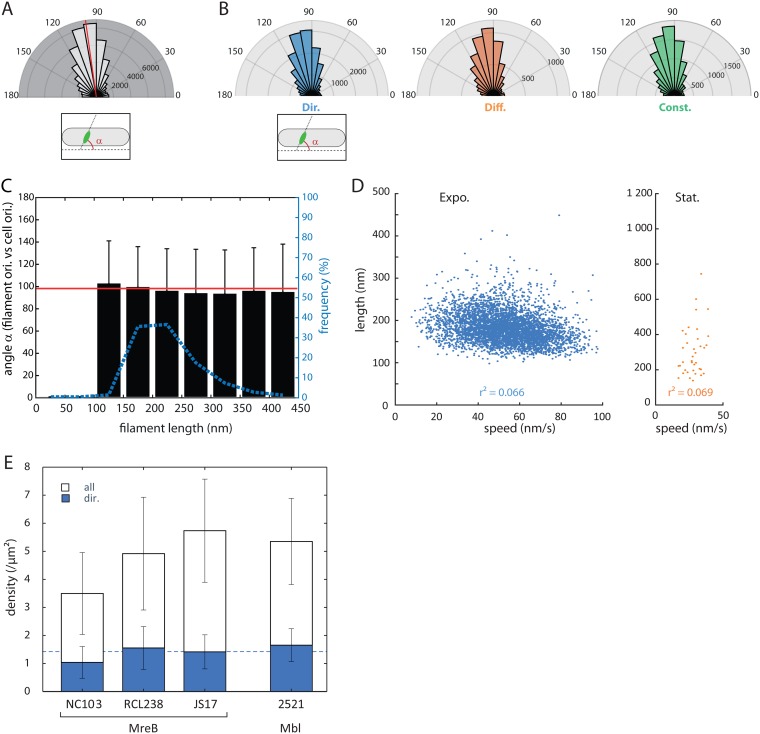
The speed of MreB filaments is not correlated with their length. (A) Distribution of angles between the long axis of the cell and the longitudinal axis (α) of GFP-MreB particles (*n* = 61,438) determined by the G-fit method from acquisitions in the SIM-TIRF mode on B. subtilis RLC238 grown to exponential phase. Filaments with a length/width ratio below 1.4 were not considered. The average is shown as a red line. (B) Same as panel A but sorted for each subpopulation of filaments: directionally moving (Dir.), diffusive (Diff.), and constrained (Const.) (*n* = 20,629, 7,459 and 12,439, respectively). (C) MreB filaments orient toward the short axis of the cell independently of their length. Distribution of the orientation of GFP-MreB filaments as a function of their length (black bars; 50 nm binning). Particle dimensions were measured by G-fit on RCL238 cells grown to mid-exponential phase observed in SIM-TIRF. Standard deviations are indicated by the error bars. Frequency (dotted blue line) shows the repartition of each subgroup (of various lengths). The average orientation of the total filament population (*n* = 61,438) is indicated as a red line. (D) Length of GFP-MreB polymers as a function of their speed, on RCL238 cells observed by SIM-TIRF, grown to mid-exponential phase (blue) and stationary phase (orange). Values are median values of the length and speed determined along each trajectory determined by the G-fit method for cells grown to exponential growth (Expo.; blue) or from kymographs for cells grown to stationary phase (Stat.; orange). Correlation coefficients for each group are indicated on the plots (*r*^2^). *n* = 4,563 (expo) and 37 (stat). (E) Density of mobile (blue) and total (white) population of particles in various strains expressing GFP-MreB (NC103, RCL238, and JS17) or Mbl-Gfp (2521). The dashed line indicates the average density of mobile filaments in the strains. The MreB-expressing strains are organized from left to right according to their (increasing) MreB levels. Analyses were performed on cells grown to exponential phase, using uTrack and MSD analysis.

Consistent with previous reports, we found that the long axis of the nanofilaments is oriented in a broad range of angles (α) relative to the long axis of the cell, around a mean angle α of 98.1° ± 37.9° (Fig. 3A). No broader distribution of angles was observed for shorter filaments (Fig. 3C), indicating that even short nanofilaments are perfectly able to orient along the cell short axis. We confirmed that directionally moving filaments travel perpendicularly to the long axis of the cell (angle γ = 89.9° ± 37.0°, Fig. S3A), and thus, that MreB nanofilaments travel in the direction of their main axis (angle β = 3.2° ± 45.3°, Fig. S3B).

10.1128/mBio.01879-18.3 FIG S3 (A) MreB particle trajectories are perpendicular to the long axis of the cell. The angle γ between the long axis of the cell and the trajectory of directionally moving GFP-MreB particles is reported here. Data are averages from populations of B. subtilis strains grown to mid-exponential phase and observed by SIM-TIRF. *n* = 4,889. (B) MreB particle trajectories are aligned toward their long axis. Here is reported the angle β between directionally moving GFP-MreB particle trajectories and their long axis, as determined by the G-fit method. Data are calculated as described above for panel A. *n* = 20,629. (C) Mobile GFP-MreB (NC103, RCL238, and JS17) and Mbl-GFP (2521) filaments are slower in stationary phase (stat.) than during exponential growth (exp.). The speeds of directionally moving filaments (upper panel) were determined using the kymograph method, and the diffusion coefficient (lower panel) were determined using uTrack and MSD analysis on acquisitions performed on cells grown to exponential (exp.) or stationary (stat.) phase. The numbers of filaments (expo/stat) on the upper panel were 108/38 (NC103), 218/37 (RCL238), 91/35 (JS17), and 78/85 (2521). The numbers of cells on the lower panel were 166/25 (NC103), 268/72 (RCL238), 168/172 (JS17), and 38/120 (2521). (D) The diffusion coefficients of randomly moving GFP-MreB particles are not correlated with their length. The lengths were determined by the G-fit method, and diffusion coefficients were determined by uTrack/MSD analysis on RCL238 cells grown to mid-exponential phase observed by SIM-TIRF. The correlation coefficient is indicated on the plot (*r*^2^). The number of filaments is 906. (E) Density of the total population of GFP-MreB (NC103, RCL238, and JS17) and Mbl-GFP (2521) filaments. The values were determined using uTrack and MSD analysis on acquisitions performed on cells grown to exponential (exp.) or stationary (stat.) phase. The numbers of cells in exponential/stationary phase were 232/108 (NC103), 297/86 (RCL238), 171/246 (JS17), and 38/142 (2521). (F) Pie chart distribution of GFP-MreB (NC103, RCL238, and JS17) or Mbl-GFP (2521) subpopulation of filaments based on their dynamic properties using uTrack and MSD analysis on exponentially grown cells. The percentages are indicated on the charts. The numbers of cells were 232 (NC103), 297 (RCL238), 171 (JS17), and 38 (2521). (G) GFP-MreB fusions have minimal impact on B. subtilis size and morphology. The wild-type strain (168) and strains expressing MreB (RCL413) or GFP-MreB (RCL238, NC103, and JS17) were grown to mid-exponential phase of growth (∼6 generations) in rich LB medium at 37°C and sampled for microscopic observation by phase-contrast and fluorescence microscopy. Each panel shows typical cells observed by phase-contrast microscopy (bar, 2 µm). The average cell width (avg.width [µm]) is reported below each panel along with the standard deviation to the mean. Widths were manually measured on fluorescent images of cell membranes stained with Nile Red (100 µM) from at least two independent experiments. Download FIG S3, TIF file, 1.3 MB. Copyright © 2019 Billaudeau et al. 2019 Billaudeau et al. This content is distributed under the terms of the Creative Commons Attribution 4.0 International license.

We next wondered whether the other subpopulations of MreB filaments display different orientations, in particular for randomly moving assemblies whose orientation was hard to predict. We found that the orientation of all three dynamic subpopulations of MreB nanofilaments is closed to that of the short axis of the cell, indicating that diffusive filaments do not realign along the direction of their trajectory while moving (Fig. 3B).

### MreB speed and dynamic properties do not depend on the nanofilament lengths.

We previously reported that in B. subtilis the speed of directionally moving MreB patches correlates with the growth rate (during active growth), suggesting that cells adapt their PG synthesis rate by modulating the velocity of the PGEM (reflected by the speed of MreB) (12). Not surprisingly, the average speed of mobile MreB strongly decreases when cells entered stationary phase, reflecting the reduced requirement for PG synthesis during this phase (Fig. S3C). However, while MreB and Mbl nanofilaments tend to be short and move fast during exponential growth, they appear longer and slower during stationary phase (Fig. S2D and S2E and Fig. S3C). It should be noted that the reduced speed in stationary phase affects not only the directionally moving fraction of the filaments but also the randomly diffusing filaments (Fig. S3C). This is reminiscent of a previously reported correlation between the speed of MreB filaments and their length (13). Similarly, we observed that during stationary phase, the strain presenting the slowest GFP-MreB fusion (JS17) also harbored the longest filaments, and reciprocally, the fastest fusion (NC103) appeared to be the shortest (Fig. S2E and Fig. S3C). However, difficulty in correlating speed and length comes from the fact that either the compared cells had different genetic backgrounds with different MreB levels (that we showed here to influence the length of the filaments) or they were in different growth states (steady versus stationary state) which, as mentioned above, could influence the speed of MreB particles, regardless of their length (12).

In order to bypass these problems and test a possible link between the length and speed of nanofilaments, we first constrained our observation to cells in steady state expressing wild-type levels of GFP-MreB (RCL238). We observed a broad distribution of filament lengths and speeds but no correlation (*r*^2^ = 0.066) between them (Fig. 3D). Likewise, length and diffusion coefficient of randomly moving nanofilaments were not correlated, which may suggest that these assemblies are not freely diffusing (Fig. S3D). Next, we measured filaments during stationary phase, using kymograph-based estimation of the lengths (Fig. 3D), but similarly, no correlation could be detected. We concluded that the speed of the particles for a given growth rate is independent of their length.

### The density of directionally moving MreB filament does not scale up with the total number of MreB assemblies.

Directionally moving MreB filaments are hypothesized to reflect the active, motioning PGEM. Because the range of strains at our disposal expressed a variety of MreB levels, we wondered how this would translate into the number of active machineries and the potential impact on B. subtilis physiology. We observed that all three strains grew at similar high rates, with JS17 having a slightly shorter generation time compared to the other two strains (Table S2). Not too surprisingly, the total density of MreB filaments (i.e., regardless of their dynamics) in exponential growth increases with the protein levels: the strain harboring the natively expressed *gfp-mreB* fusion displayed the lowest number of filaments and the merodiploid strain expressing the inducible fusion displayed the highest (Fig. 3E and Fig. S3E). However, the fraction of directionally mobile filaments did not increase proportionally to the total number of assemblies (Fig. S3F). The density of processive filaments remained constrained in a range of 1 to 1.5 µm^−2^ (Fig. 3E), a similar value to that observed for GFP-Mbl. This suggests that the number of mobile filaments is not limited by the availability of MreB, at least in RCL238 and JS17, and reinforce the hypothesis that static filaments are excess material playing the role of a buffering system, waiting for other components to be available.

10.1128/mBio.01879-18.5 TABLE S2 Growth rates of B. subtilis strains used in this study. Download Table S2, TIF file, 0.1 MB. Copyright © 2019 Billaudeau et al. 2019 Billaudeau et al. This content is distributed under the terms of the Creative Commons Attribution 4.0 International license.

## DISCUSSION

### Nanofilaments are the native form of B. subtilis MreB.

Here we revisited the long debated question of the structure of MreB assemblies during active growth and found new evidence that *in vivo* MreB does not form micron-long assemblies in B. subtilis when expressed at native levels. Instead, MreB forms on average <200 nm-long nanofilaments during active growth. Thus, micron-long assemblies are not required for normal growth, although they can be induced by increasing the MreB levels during stationary phase with minimal perturbations. It is interesting to note however that in E. coli MreB is reported to form longer filaments (∼500 nm), using a fusion to msfGFP considered to be the less perturbative available in this species (29, 30). Although this difference may reflect the different strategies of PG synthesis deployed in each organism (12), it would be interesting to assess whether levels of MreB, despite its expression from its natural promoter, are not affected in this species by the presence of the fluorescent tag.

### Size does not matter.

**FIG 4:**
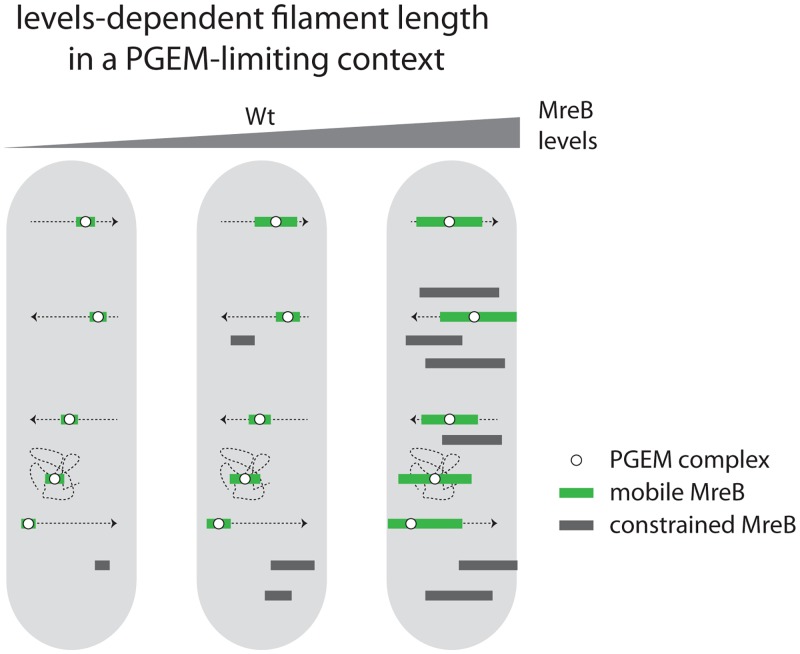
The length and the total number of MreB filaments, but not the number of directionally moving filaments, scale up with MreB cellular levels. During exponential growth, in cells expressing wild-type levels of MreB (middle panel), the majority of filaments are circumferentially moving (green boxes with straight arrows) while other MreB filaments exhibit a random motion (green boxes with entangled lines) or remain constrained (grey boxes). At lower MreB concentrations (left panel), filaments are shorter and their total number decreases, but the number of directionally moving filaments remains unchanged. At concentrations above native levels (right panel) filaments are longer and more abundant. The number of constrained filaments increases while the number of directionally moving filaments remains again unchanged. It is hypothesized that the PG elongation machineries (PGEMs) containing the enzymatic activity for CW elongation are associated only to mobile MreB filaments.

From our results, it appears that B. subtilis has a large resilience to a broad range of MreB levels or filament size, since the three strains grow mainly as rod-shaped bacteria (see Fig. S3G in the supplemental material). It seems that having longer than normal filaments ranging from the natural <200 nm length on average to >1 µm long has minimal if any impact on the physiology of the cell. A possible reason for this resilience is that longer structures are observed when cells exit exponential growth, which may limit the impact of any putative perturbations from the formation of extended filaments. The other probable cause is that the length of MreB polymers does not affect the speed or behavior of the filaments, hence the CW synthesis and finally the growth rate. This advocates against the theory in which the speed of rotating MreB filaments would scale up with their length due to a cooperative effect of associated PGEMs, then would decrease above a threshold due to a “tug-of-war” competition between oppositely facing PGEM (13). However, it is likely that reducing the size of MreB filaments below some threshold will finally impact its ability to align correctly and to maintain the rod shape according to the recent finding of Hussain et al. (16) that MreB would sense the cell curvature and align along the highest curve. The slight increase in cell width and more frequent misshaped cells observed in NC103 advocate for this (Fig. S3G). A final cause of this resilience to filament length could be that the number of synthetic enzymes (PGEM) associated with a filament does not scale up proportionally to the size of the MreB polymers. We previously made rough estimations of the number of machines required to double B. subtilis (and E. coli) surface during exponential growth based on the growth rate, cell size, and density and speed of the assemblies (12). The striking result was that each filament would insert only a few PG strands requiring a very limited number of PG synthetic enzymes. Our refined results using SIM-TIRF observation have now significantly increased the density of mobile assemblies, decreasing the estimated size of the PG bands inserted per filament to 4 to 5 nm, which is the estimated size of a single PG strand. If each filament contains a single set of PG synthetic enzymes, it argues against the possibility that MreB could synchronize machines along the side wall, even more against a “tug-of-war’” model. Although these estimations should be taken with care, they suggest that the number of PGEMs is limited compared to available MreBs and that another factor dictates the number of available machines (hence mobile MreB filaments) at any given time. The observation of a limited density of mobile filaments, regardless of the total density of filaments or protein levels, also advocates for this. This in turn explains the resilience to broad MreB levels: size would not matter much if the structures extend because they are not correlated with the number of machines, the filament would just grow longer as the MreB concentration rises. Thus, and without excluding the possibility that other factors may contribute to the polymerization of MreB, the cell would also dispose of two buffering systems for MreB overflow by forming inactive polymers and extending the polymer size (Fig. 4).

### On MreB function.

The cellular function of MreB and the reason it forms polymers have remained elusive until the recent proposal that it would align to the highest curvature of the cell (the short axis) providing a directionality to the PGEM movement (16). Consistent with this model, our results show that directionally moving MreB assemblies align along their trajectories (hence along the short axis of the cell) regardless of their length, suggesting that this orientation is an intrinsic property of the filaments. In such a model, MreB polymerization would not need to be highly dynamic, in contrast to its related actin filaments, matching the previously reported absence or very low turnover of monomers (in the filament during the time course of their trajectory across the cell) (9) and mild defects of *mreB* alleles with mutations in their presumed active site (e.g., MreB^D158A^) (9, 10, 31). This would provide a purpose to its polymerization, but it does not exclude additional functions for the protein such as recruiting and potentially activating the PGEM complex or maintaining its structural integrity.

## MATERIALS AND METHODS

### General methods, bacterial strains, and growth conditions.

Basic methods for growth and manipulation of B. subtilis have been described extensively elsewhere (32). B. subtilis was grown at 37°C in rich lysogeny broth medium (LB) supplemented with 25 mM MgSO_4_, supplemented with 0.5% xylose for promoter induction when appropriate. Antibiotics were used only for precultures at the following concentrations: chloramphenicol, 5 μg·ml^−1^; kanamycin, 10 μg·ml^−1^; spectinomycin, 100 μg·ml^−1^; erythromycin, 1 μg·ml^−1^. B. subtilis strains used in this study are listed in Table S1 in the supplemental material.

### Western blot analysis.

Samples were collected at an optical density at 600 nm (OD_600_) indicated on figures, and cell extracts were prepared from cultures grown in LB (supplemented with 20 mM MgSO_4_ and 0.5% xylose if required) and loaded on polyacrylamide-SDS gels as previously described (33). Blots were performed, and proteins were detected as described in reference 33 using polyclonal antibodies against MreB or GFP (1:10,000).

### TIRFM and TIRF-SIM.

For all acquisitions, cells were first grown as overnight precultures at 30°C in LB and with maximum aeration in the presence of selective pressure, then diluted at a maximum OD_600_ of 0.005 and grown at 37°C. Imaging was performed when cells reached early exponential phase (OD_600_ of ∼0.1 to 0.2) and stationary phase (OD_600_ of ∼2 to 3). One microliter of the liquid culture was then spotted onto a freshly made, prewarmed, thin agarose pad (1% in LB), topped by a coverslip and immersion oil, and mounted immediately in the temperature-controlled microscope stage. All acquisitions were done at 37°C, within 30 min after taking the sample, with an exposure time of 100 ms and interframe intervals of 1 or 2 s over 1 or 2 min.

Time-lapse TIRFM movies were taken on a previously described setup (12). TIRF-SIM imaging was performed at the Advanced Imaging Center (AIC) (Janelia Research Campus, VA, USA) as previously described (28). Briefly, excitation patterns were produced using a phase-only spatial light modulator (BVO AHWP3; Bolder Vision Optik). A mask system was used to select specifically the ±1 diffraction orders, which were then focused onto the back focal plane of an Olympus Plan-Apochromat 100× 1.49NA objective. For optimal interference contrast in the sample, the polarity of the light was rotated to match the angle of the pattern using a liquid crystal variable retarder (LC, Meadowlark, SWIFT) and wave plates. Emissions were collected using interference filters and imaged onto a pair of sCMOS cameras (Orca Flash 4.0 v2 sCMOS; Hamamatsu). Samples were maintained at 37°C using a stage-top incubator (H301; okolabs, Naples, Italy). SIM reconstructed images and average TIRF images were processed from nine raw acquisitions (three translations × three rotations), as described in reference 28. The experimental resolution limit at 488 nm was estimated to be 114.37 ± 16 nm (*n* = 198 beads), using 40-nm fluorescent beads.

### Single particle tracking and patch density quantification of MreB.

Single particle tracking (SPT) was performed mainly as previously described (12), using u-track 2.1.3 software (MATLAB-based suite [34, 35]), except that the entire field of views was processed before regions of interest (ROI), defined on single cells, were selected. During SPT analysis, comet detection approach stands on difference of Gaussian filtering (σ_1_=1.5 pixels for low-pass and σ_2_=4 pixels for high-pass Gaussian standard deviation [SD] in exponential phase and σ_1_=1.0 pixels and σ_2_=9 pixels in stationary phase) followed by watershed segmentation (minimum threshold of 6 and 5 SD of image intensity in exponential and stationary phase, respectively, with a step size of 1 SD). Then, SPT was performed by linking localizations close to each other on consecutive frames. No missing link was allowed in the tracking (maximal gap to close = 0), and splitting or merging of tracks was also not allowed, which meant that the linking cost during reconnection was mainly dependent on patch-to-patch distance. The Brownian search radius was set between 0 and 3 pixels.

TIRF images generated from raw SIM acquisitions were used to define ROI as follows. First, each frame of temporal sequence was segmented, followed by a maximum intensity projection and morphological closing with a disk as a structuring element (radius of 5 pixels) were sequentially applied to obtain a binary mask of cell areas. Finally, cells were selected for analysis and quantification (area, long-axis orientation, patch dynamics within each single cell).

### Classification of MreB filament dynamics.

Particle dynamics were categorized based on MSD analyses as previously described (12). Only trajectories with a minimal duration of six consecutive frames were considered.

Each patch detected was assigned to a class (directed, diffused, constrained, or unclassified) for all frames of its corresponding movie, allowing us to calculate instantaneous patch density and mobile fractions per frame, and thus, to estimate averages at the single cell level. Speed (ν) was quantified using solely patches exhibiting directed motion, and diffusion coefficient was extracted from random diffusion trajectories only. The trajectory orientation of each directed patch was estimated from the slope of linear regression applied on all temporal positions.

### Determination of filament dimensions and orientations by 2D Gaussian fit.

The dimensions of the particles detected with u-track and their orientation relative to their trajectories were quantified using 2D anisotropic Gaussian fit as follows:

$$f\left(x,y\right)=B_{0}+I_{0}\text{exp}\left[-{\left(\frac{\left(x-x_{0}\right)\cos\theta +\left(y-y_{0}\right)\sin\theta }{\sigma_{1}}\right)}^{2}-{\left(\frac{-\left(x-x_{0}\right)\sin\theta +\left(y-y_{0}\right)\cos\theta }{\sigma_{2}}\right)}^{2}\right]$$

where *B*_0_ and *I*_0_ are the background intensity and amplitude of the Gaussian centered at (*x*_0_,*y*_0_) with lateral widths (σ_1_,σ_2_) and θ is the orientation of the Gaussian in image coordinates. The length and width of filaments were respectively defined as the maximum and minimum values of full width at half-maximum $\left(2\sigma_{i}\sqrt{2\log2},\;i=1,2\right)$ and filament orientation along length axis. In order to prevent quantification errors, filament orientation was performed only for isolated filaments (alone inside disk with a radius of 5 pixels) with minimal width of 80 nm and with an aspect ratio (defined as length/width) above 1.4.

### Determination of long structure dimensions based on kymograph.

The dimensions of structures longer than the field of view were determined based on kymograph using a macro developed on Fiji specifically for this analysis (Fig. S2A). Profile lines (width of 1 pixel) were drawn on filaments spatially separated from other filaments and exhibiting directional movements roughly perpendicular to the long axis of the cell. Maxima were automatically detected along the kymograph and fitted using linear function to quantify filament velocity (*v*). Each filament length was estimated as *v*×*t_vis_* where *t_vis_* is the average duration during which the filament is visible on each kymograph position. Only filaments entirely visible (appearing and disappearing) during the window of acquisition (and excluding the first and last five frames for certainty) were kept.

### Choice of method to measure structure dimensions.

The choice between the 2D Gaussian fit (G-fit) and the kymograph-based method to measure MreB filament lengths depends on the average size of the structures. For short structures, both the kymograph and the G-fit methods can be used as they give similar length measurements (Fig. S2B). However, the kymograph is far more time-consuming and is restricted to the analysis of directionally moving particles; hence, G-fit is favored under this condition. For longer structures, two problems occur with the G-fit method: objects can extend beyond the visible area of the cell (above the half diameter), and objects extending close to this limit will present a decreased intensity due to the larger distance between the coverslip and the fluorescent moiety, due to the limited depth penetration in the TIRF mode. This can be shown by simulating directed moving structures whose sizes varied from the light diffraction limit (∼110 nm) to filaments larger than the cell diameter (1,200 nm) (Fig. S2C). For this, time-lapse acquisition is simulated using SIM-TIRF images of 40 nm fluorescent beads by applying 1 pixel (40.5 nm) translation between each frame, while the time between two consecutive frames was set at 1 s. Then, using average intensity projection of *k* images, the initial size of fluorescent beads is elongated by additional length of (*k* − 1) × pixel (*k* = 1 to 30 in our simulations) and binary mask is then applied to mimic cell diameter extension (1 µm). Next, we can address the influence of TIRF illumination on filament length quantification by applying a correction factor to the intensity exp(−*z/d*), allowing us to take into account penetration depth in TIRF (*d*=250 nm) and height *z* of filament relative to the coverslip, defined as *z*=*r*[1-cos⁡(sin^-1^⁡(*dx*/*r*))], where *r* is the cell radius and *dx* is the lateral distance separating the filament position from the contact point of the cell with the coverslip. This allows us to determine the limits of length quantification based on Gaussian fit on 1D intensity profile for partially observed objects and how TIRF illumination profile can impact measured lengths. On the basis of these simulations, we confirm that length quantification based on kymograph is a proper method to recover the true size of filaments without bias on a full range of simulated lengths (black in Fig. S2C). In contrast, lengths of filament measured using Gaussian fit on 1D intensity profiles are underestimated as soon as the measured objects are partially observed (red) or when intensity decrease provides an apparent size smaller than the true size (magenta).

For this reason, the kymograph is a preferred solution to measure extended filaments.
