# Supplementary figures and images for: MreB Forms Subdiffraction Nanofilaments during Active Growth in Bacillus subtilis
Source: mBio. 2019 Jan 29;10(1):e01879-18. doi: 10.1128/mBio.01879-18 (PMC6355991; doi:10.1128/mBio.01879-18)

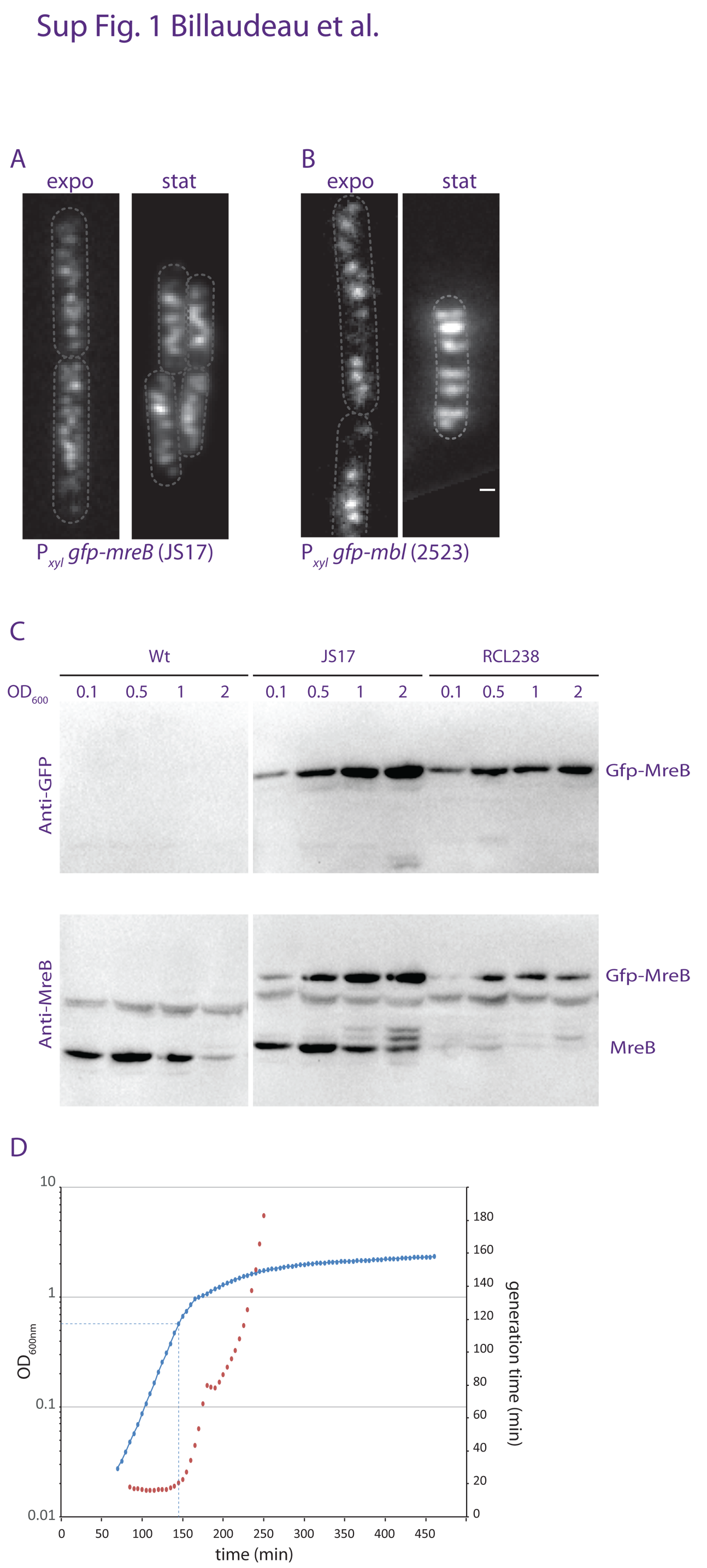

Supplement: FIG S1 [file mBio.01879-18-sf001.tif]

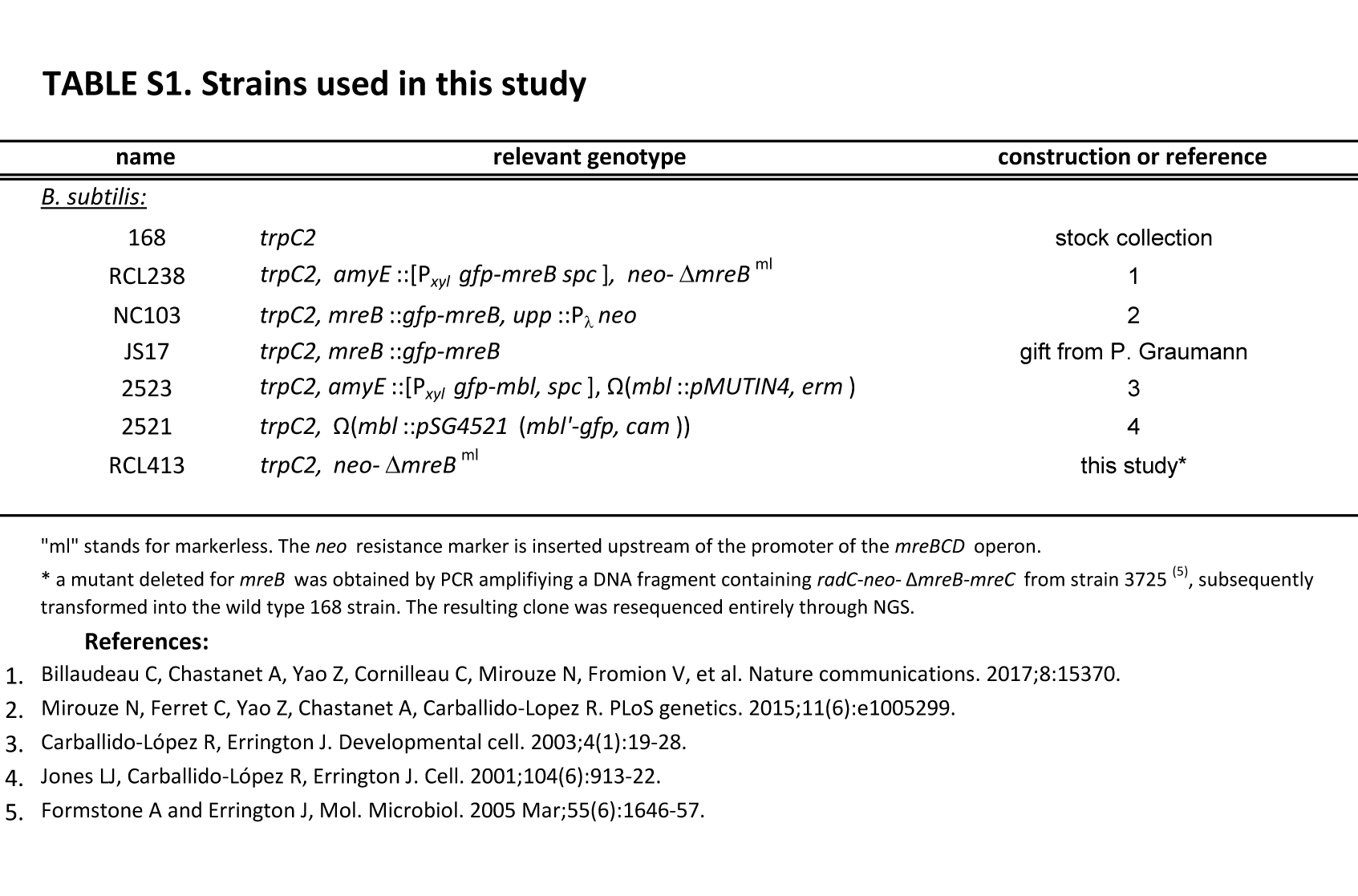

Supplement: TABLE S1 [file mBio.01879-18-st001.tif]

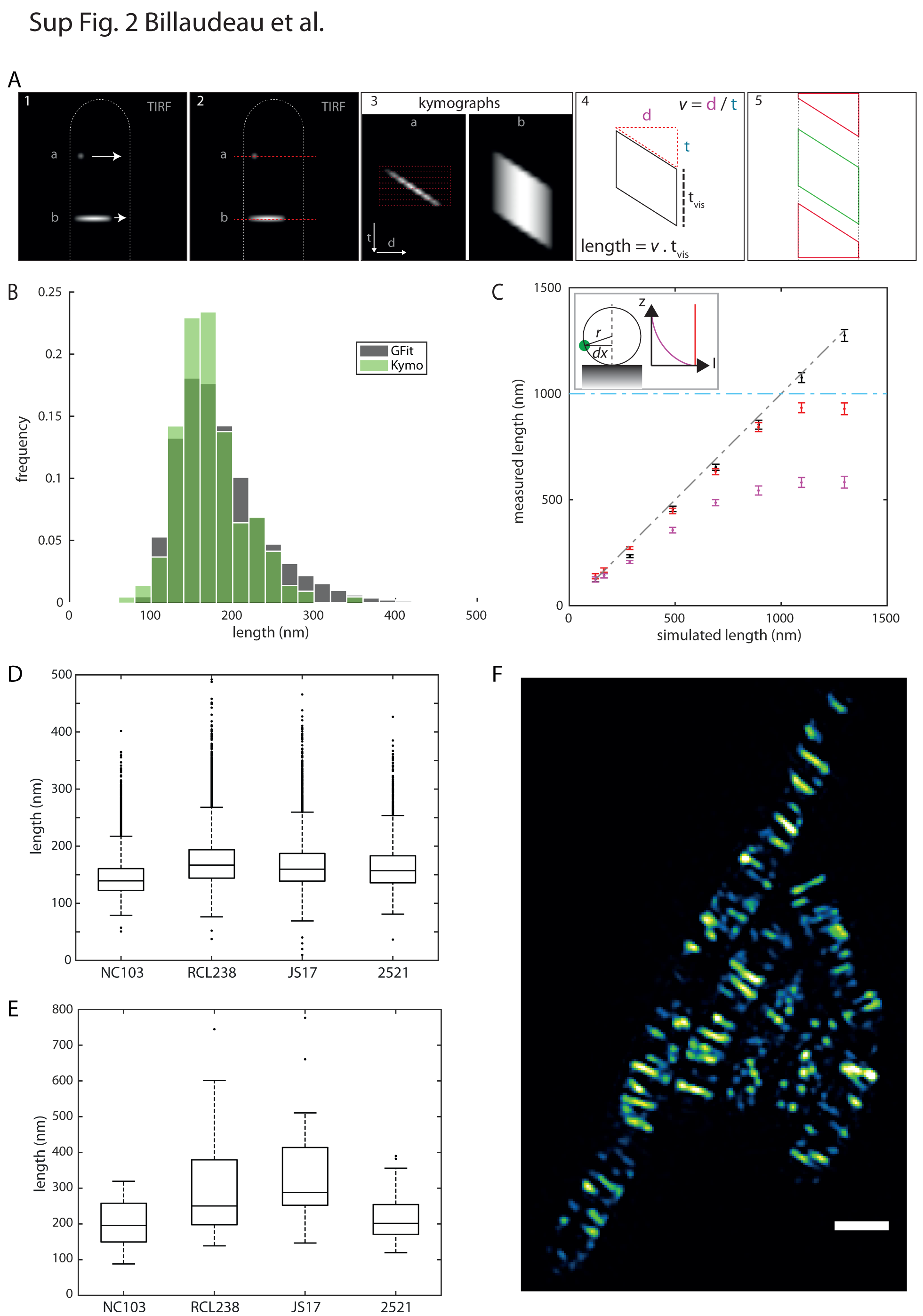

Supplement: FIG S2 [file mBio.01879-18-sf002.tif]

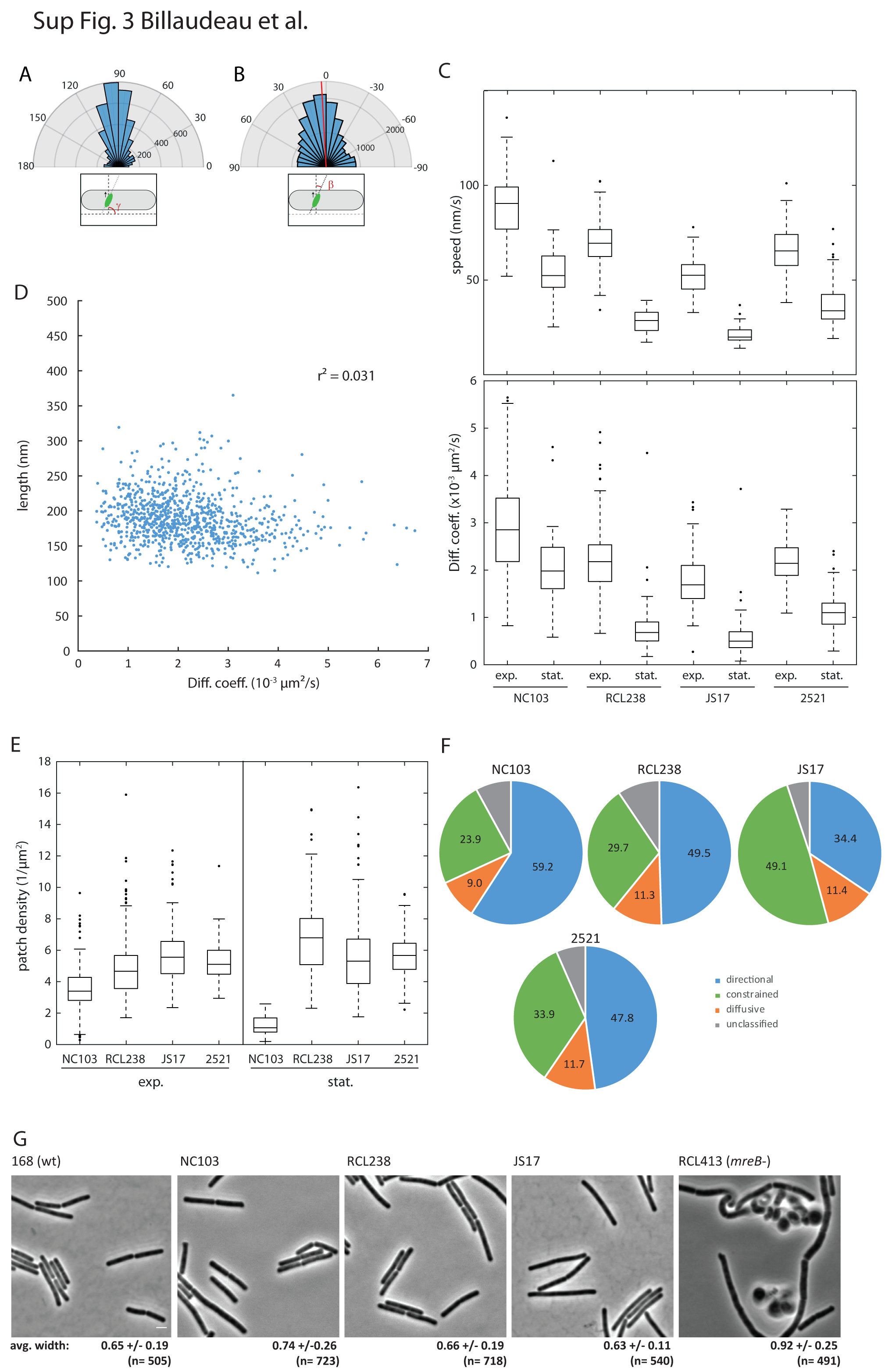

Supplement: FIG S3 [file mBio.01879-18-sf003.tif]

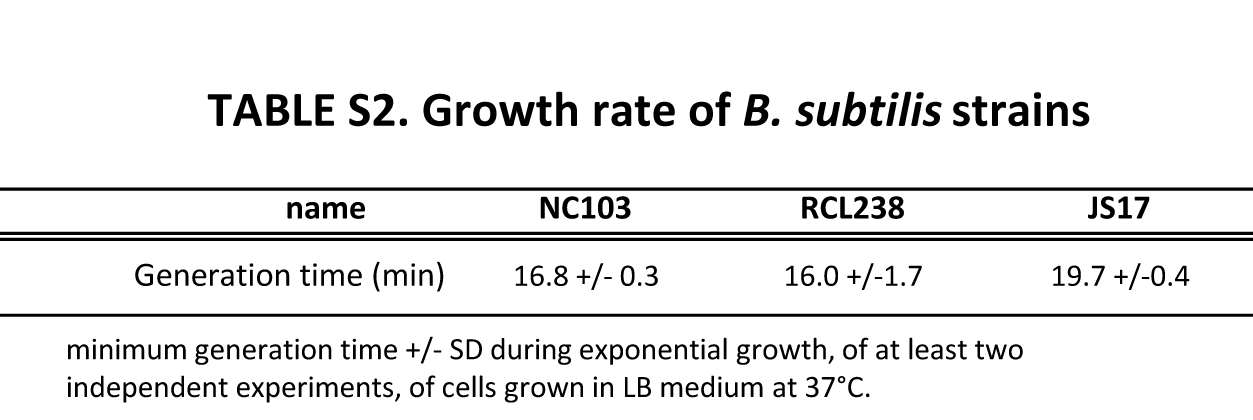

Supplement: TABLE S2 [file mBio.01879-18-st002.tif]
